# Plasma cell-free DNA in patients needing mechanical ventilation

**DOI:** 10.1186/cc10357

**Published:** 2011-08-12

**Authors:** Marjatta Okkonen, Päivi Lakkisto, Anna-Maija Korhonen, Ilkka Parviai-nen, Matti Reinikainen, Tero Varpula, Ville Pettilä

**Affiliations:** 1 Division of Anaesthesia and Intensive Care Medicine, Department of Surgery, Helsinki University Central Hospital, Haartmaninkatu 4, PO Box 340, 00029 HUS, Helsinki, Finland; 2 Department of Clinical Chemistry, Helsinki University Central Hospital, Haartmaninkatu 4, PO Box 340, 00029 HUS, Helsinki, Finland; 3 Minerva Research Institute, BIOMEDICUM Helsinki 2U, Tukholmankatu 8, 00290 Helsinki, Finland; 4 Division of Intensive Care, Kuopio University Hospital, Puijonlaaksontie 2, PO Box 1777, 70211 Kuopio, Finland; 5 Department of Intensive Care, North Karelia Central Hospital, Tikkamäentie 16, 80210 Joensuu, Finland

## Abstract

**Introduction:**

Concentrations of plasma cell-free DNA are increased in various diseases and have shown some prognostic value in many patient groups, including critically ill patients. Pathophysiological processes behind the need for mechanical ventilation and the treatment itself could raise plasma levels of cell-free DNA. We evaluated levels of plasma cell-free DNA and their prognostic value in patients needing mechanical ventilation.

**Methods:**

We studied prospectively 580 mechanically ventilated critically ill patients. Blood samples were taken at study admission (Day 0) and on Day 2. Plasma cell-free DNA concentrations were measured by real-time quantitative PCR assay for the β-globin gene and are expressed as genome equivalents (GE)/ml.

**Results:**

Median (interquartile range, IQR) plasma cell-free DNA concentration was 11,853 GE/ml (5,304 to 24,620 GE/mL) at study admission, and 11,610 GE/mL (6,411 to 21,558 GE/mL) on Day 2. Concentrations at admission were significantly higher in 90-day non-survivors than survivors, 16,936 GE/mL (7,262 to 46,866 GE/mL) versus 10,026 GE/mL (4,870 to 19,820 GE/mL), *P *< 0.001. In a multivariate logistic regression analysis plasma cell-free DNA concentration over 16,000 GE/ml remained an independent predictor of 90-day mortality (adjusted odds ratio 2.16, 95% confidence interval CI 1.37 to 3.40). Positive likelihood ratio of plasma cell-free DNA at admission for the prediction of 90-day mortality was 1.72 (95% CI 1.40 to 2.11).

**Conclusions:**

Plasma levels of cell-free DNA were significantly higher in non-survivors than survivors. Plasma DNA level at baseline was an independent predictor of 90-day mortality. However, its clinical benefit as a prognostic marker seems to be limited.

## Introduction

Cell-free DNA is detected in blood in many diseases, but also in healthy individuals. Cell-free DNA can originate from necrotic cells or apoptotic processes, and active release of DNA fragments from living cells has also been described [1]. The exact mechanism of DNA occurrence in blood, however, is not fully understood. Knowledge about the elimination of cell-free DNA from blood is inadequate [2], but available data suggest that more than one mechanism is involved in its clearance. In a recent meta-analysis, based on 39 studies, cell-free DNA concentrations up to 4,000 genome equivalents (GE)/ml in healthy controls were reported [3]. Higher levels have been measured in different pathophysiological states: in malignancies [4], sepsis [5,6], acute pancreatitis [7,8], trauma [9], stroke [10], myocardial infarction [11], and abdominal pain [12]. Plasma cell-free DNA may have prognostic value in many acute clinical situations: patients with sepsis [6], acute coronary syndrome (ACS) [13], trauma [9], pancreatitis [7], and in patients after cardiac arrest [14].

The need for mechanical ventilation (MV) is a consequence of diverse pathophysiologic conditions of both pulmonary and extrapulmonary origin leading to impaired oxygenation and/or ventilation or a need to secure the airway and support ventilation because of impaired consciousness. Deteriorated oxygenation can *per se *cause tissue hypoxia and, thus, damage to cells. In addition, pathophysiologic processes leading to the need of MV are potentially accompanied with cell death and the release of DNA to circulation. Furthermore, MV itself may cause damage to lung tissue [15] and theoretically induce release of DNA to circulating plasma. Association between injurious ventilation and increased remote organ apoptosis as well as increased type 2 cell necrosis in lungs has been shown in an animal study [16]. However, no large-scale human studies regarding plasma cell-free DNA in patients needing MV have been published.

The aim of our study was to assess plasma cell-free DNA concentrations and their prognostic value on 90-day mortality in a large observational multi-centre study on mechanically ventilated critically ill patients.

## Materials and methods

We conducted a prospective, epidemiological cohort study on patients needing MV in 25 Finnish intensive care units (ICU) during an eight-week period (from 16 April to 10 June 2007) (The FINNALI study). In this study we pros-pectively evaluated all patients (≥16 years) treated in ICUs. The study design and epidemiological results have been published previously [17]. In brief, all patients treated with respiratory support for more than six hours either with invasive or non-invasive interface were included in the study. The local ethics committees approved the study. Informed consent from patients or surrogates was required for laboratory samples.

We registered demographic data, underlying risk factors for the need of MV, physiological and ventilatory data at the study admission, and medications. The clinical report form (CRF) data were reported as an attachment to the Finnish Intensive Care Consortium routine dataset which includes data about reason for admission, severity scorings (Simplified Acute Physiology Score (SAPS) II [18] and Sequential Organ Failure Assessment (SOFA) score [19]), and hospital outcome. Acute lung injury (ALI) and acute respiratory distress syndrome (ARDS) were defined according to the American-European Consensus Conference (AECC) criteria [20]. Patients were divided to those who had infection and those who did not by Acute Physiology and Chronic Health evaluation (APACHE) III, ICD10-diagnoses (International Statistical Classification of Diseases and Related Health Problems), and the presence of pneumonia or sepsis as an underlying risk factor during the preceding 48 hours. Data collection was accomplished with an Internet-based interface. Mortality data were obtained from Statistics Finland [21]. This study is a prospective sub-study evaluating plasma cell-free DNA in the FINNALI cohort.

### Blood sampling

Two blood samples were drawn: first at the time of study inclusion (Day 0, sample A) and second in the morning of Day 2 (sample B) to 10 ml heparin containing tubes. Plasma was separated as soon as possible by centrifugation at 1,500 *g *for 15 minutes and transferred to acid-handled plastic tubes, which were stored at a minimum of -20°C in each hospital until the study completion. All samples were collected at Helsinki University Central Hospital and stored at -80°C.

### Quantification of plasma cell-free DNA

DNA extraction and quantification of plasma cell-free DNA were performed as described earlier [6,22]. Briefly, plasma samples were centrifuged at 16,000 *g *for 10 minutes before DNA extraction to remove any residual cells [23]. DNA was extracted using the QIAamp DNA Blood Mini Kit (Qiagen, Hilden, Germany) according to the "blood and body fluid protocol", and plasma cell-free DNA was measured by real-time quantitative PCR assay for the β-globin gene. A 10-fold serial dilution of human genomic DNA (Roche Diagnostics GmbH, Mannheim, Germany) was used as a standard curve in the PCR assay. Results are expressed as genome equivalents (GE)/ml; 1 GE equals 6.6 pikograms of DNA. In this study, we considered plasma cell-free DNA concentration of 4,000 GE/ml as an upper limit of normal range, value obtained from a recent meta-analysis [3].

### Statistics

Data are presented as median and interquartile ranges (IQR, 25^th ^and 75^th ^percentiles) or numbers and percentages. The primary endpoint was 90-day mortality. For comparisons of non-parametric data between survivors and non-survivors, we used Mann-Whitney U-test. We assessed the differences in plasma cell-free DNA concentrations between different strata according to the severity of oxygenation impairment, as reflected by the baseline PaO_2_/FiO_2_-ratio. We also compared non-pulmonary SOFA scores between these strata to find out whether there was a correlation between impaired oxygenation and failure of other organ systems. We first used the Kruskal-Wallis test and then the *post hoc *Mann-Whitney test with *P *< 0.01 considered as significant due to multiple comparisons. The Spearman's rank correlation test was performed to evaluate the association between plasma DNA at admission, baseline pH, renal SOFA points at Day 1, SAPS II score, baseline PaO_2_/FiO_2_-ratio, and baseline tidal volume per predicted body weight. We analyzed the receiver operating characteristic (ROC) curve for plasma cell-free DNA at admission for 90-day mortality prediction. For calculations of sensitivity, specificity and positive likelihood ratios (with 95% confidence intervals, CI) of plasma cell-free DNA for prediction of 90-day mortality we used two cutoff points, one obtained from the earlier study [6] and one from the present study by Youden's method [24]. For identification of independent predictors of 90-day mortality we performed a multivariate backward logistic regression analysis. Variables included in the analysis were: plasma cell-free DNA over the best cutoff value at admission, present infection, and all variables found to be independent predictors for 90-day mortality in the FINNALI study (SAPS II score minus oxygenation, chronic heart disease, suspected aspiration, intoxication, and baseline PaO_2_/FiO_2_-ratio). We also made another multivariate backward logistic regression analysis with otherwise same variables, but plasma cell-free DNA was included as a categorical variable divided to arbitrary intervals of 5,000 GE/ml. We constructed Kaplan-Meier curves of 90-day survival according to plasma cell-free DNA at admission. Finally, we performed a subgroup analysis excluding cardiac surgery patients and including only patients with an emergency admission and invasive ventilation exceeding 24 hours. We compared the plasma DNA levels of this subgroup to the rest of the study patients by the Mann-Whitney test. In all analyses, *P *< 0.05 was considered as significant.

## Results

### Study patients

**Figure 1:**
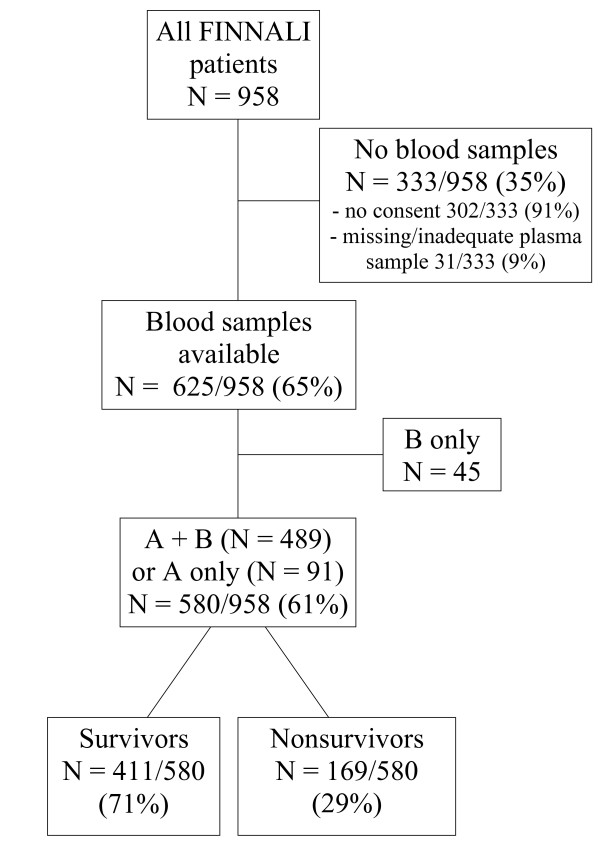
**Flowchart of study patients**. **(A) **Blood sample at study admission (Day 0); (**B) **blood sample at Day 2.

The study flowchart is presented in Figure 1. Of 958 patients in the FINNALI study, all 580 patients (61%) with consent given for laboratory samples and at least sample A (at study admission) available were included to this study. Sample A was drawn six hours (median, IQR 6 to 8 hours) and sample B 42 hours (median, IQR 38 to 47 hours) after the start of ventilatory support. Study patients were older (65 vs. 60 yrs.) and more often elective admissions (17 vs. 9%), but did not differ significantly from the rest of the FINNALI study patients in gender, hospital or 90-day mortality, SAPS II score, SOFA score at 24 hours, operative status, or ventilatory support time (data not shown).

Characteristics of the study patients divided into subgroups are presented in Table 1, and baseline physiological characteristics in Table 2. Predisposing factors for the need of MV among 580 patients were: surgery in 229 (39%), decreased level of consciousness in 146 (25%), cardiac insufficiency in 128 (22%), suspected aspiration in 58 (10%) and witnessed aspiration in 29 (5%), sepsis in 82 (14%), pneumonia in 68 (12%), other respiratory infection in 60 (10%), intoxication in 34 (6%), massive transfusion in 31 (5%), severe trauma in 29 (5%), acute pancreatitis in 20 (3%), and neuromuscular disease in 16 (3%) patients. Invasive ventilation was used in 506 of 580 study patients (87%). Of 229 postoperative patients, 99 (43%) were elective admissions, mostly (64 patients, 65%) for cardiac surgery. Hospital and 90-day mortalities were 134 (23%, 95% CI 20 to 27%) and 169 (29%, 95% CI 25 to 33%) of the 580 patients, respectively.

**Table 1:** Characteristics of the study patients

	All patients N = 580	Postoperative patients N = 229	Non-operative patients	
			Infection N = 124	No infection N = 227
Age	61 (51, 74)	69 (56, 76)	63 (55, 73)	62 (48, 74)
Male, n (%)	382 (66)	150 (66)	85 (69)	147 (65)
SAPS II	42 (30, 55)	33 (26, 46)	42 (34, 54)	51 (36, 63)
SOFA at 24 hours	8 (5, 10)	7 (5, 9)	7 (5, 10)	8 (6, 10)
Max SOFA total	9 (6,12)	9 (6, 11)	10 (6, 14)	9 (6, 12)
Invasive ventilation, days	2 (1, 4)	2 (1, 4)	2 (0, 5)	2 (1, 4)
Length of stay, ICU, days	3.2 (1.7, 6.8)	2.9 (1.7, 7.1)	5.0 (2.6, 7.7)	2.7 (1.6, 5.8)
Hospital mortality, n (%)	134 (23)	33 (14)	37 (30)	64 (28)
90-day mortality, n (%)	169 (29)	44 (19)	44 (36)	81 (36)

Data are presented in numbers (percentages) or median (interquartile range).

SAPS, Simplified Acute Physiology Score; SOFA, Sequential Organ Failure Assessment; ICU, intensive care unit

**Table 2:** Physiological characteristics of the study patients at baseline

	All patients (N = 580)
**PaO_2_/FiO_2_-ratio**	247 (172, 338)
**Tidal volume/Predicted body weight**	8.6 (7.5, 9.9)
**PEEP**	6 (5, 8)
**MAP**	76 (67, 86)
**CVP**	9 (7, 13)
**MV (intubated)**	485 (84%)
**Vasopressors**	304 (52%)

Data are presented in numbers (percentages) or median (interquartile range).

CVP, central venous pressure; MAP, mean arterial pressure; MV, mechanical ventilation; PEEP, positive end expiratory pressure

### Concentrations of plasma cell-free DNA

In the 580 patients, median (IQR) plasma cell-free DNA concentration was 11,853 GE/mL (5,304 to 24,620 GE/mL) in sample A and 11,610 GE/mL (6,411 to 21,558 GE/mL) in sample B. Of the 580 patients, 428 (82%) had a plasma cell-free DNA concentration at admission (sample A) that was higher than normal (4,000 GE/ml). In postoperative patients (N = 229), the median plasma cell-free DNA concentration (IQR) at admission was 11,436 GE/ml (5,587 to 20,379 GE/ml); in non-operative patients (N = 351), the median (IQR) concentration at admission was 12,156 GE/ml (4,938 to 27,292 GE/ml) (*P = *0.36). The change in plasma cell-free DNA concentration from sample A to sample B was different in post-operative (rising) and non-operative (descending) patients (*P *< 0.01). Non-operative patients with infection (N = 124) had significantly higher plasma cell-free DNA concentration at admission than those without infection (N = 227): median (IQR) 15,859 GE/ml (7,882 to 32,887 GE/ml) vs. 9,500 GE/ml (4,142 to 23,386 GE/ml) (*P *< 0.001), respectively. In postoperative patients the corresponding values did not differ between patients with infection (N = 55) or without it (N = 174): median (IQR) 12,560 GE/ml (7,278 to 28,662 GE/ml) vs. 9,595 GE/ml (5,264 to 19,667 GE/ml) (*P *= 0.053), respec-tively.

**Figure 2:**
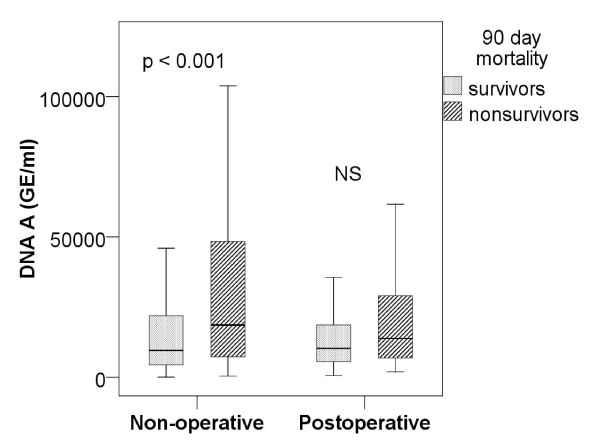
**Plasma cell-free DNA at admission in 90-day non-survivors and survivors according to operative status**. Data are presented as median values (lines) with 25^th ^and 75^th ^(boxes) and 5th and 95th percentiles (whiskers). Sample A obtained at admission. GE, genome equivalents; NS, non-significant.

The median (IQR) plasma cell-free DNA concentration at admission was significantly higher in 90-day non-survivors than survivors: 16,936 GE/mL (7,262 to 46,866 GE/mL) vs. 10,026 GE/mL (4,870 to 19,820 GE/mL) (*P *< 0.001). In the sample B the difference was not significant: median (IQR) 12,157 GE/mL (6,722 to 25,933 GE/mL) in 90-day non-survivors vs. 11,556 GE/mL (5,962 to 19,735 GE/mL) in survivors (*P *= 0.094). The difference at admission was statistically significant in non-operative patients, but not in postoperative patients (Figure 2).

**Figure 3:**
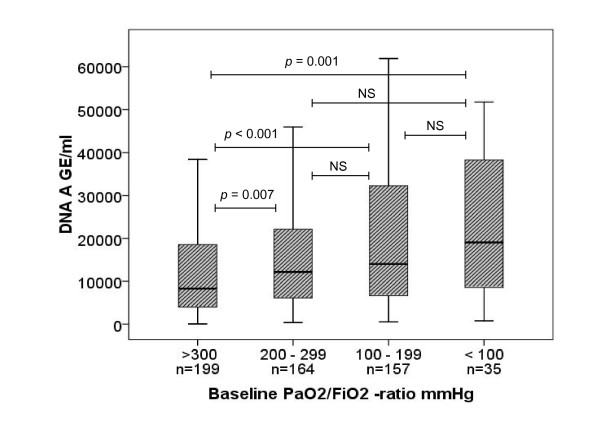
**Plasma cell-free DNA at admission according to baseline PaO_2_/FiO_2_-ratio based on SOFA cut-offs**. Data are presented as median values (lines) with 25^th ^and 75^th ^percentiles (boxes) and 5^th ^and 95^th ^percentiles (whiskers). Sample A obtained at admission. Comparison between groups by Kruskal-Wallis test (*P *< 0.001). *Post hoc *analyses between groups by Mann-Whitney test (*P *< 0.01 considered significant due to multiple comparisons). GE, genome equivalents; NS, non-significant; SOFA, Sequential Organ Failure Assessment.

Plasma cell-free DNA concentrations according to baseline PaO_2_/FiO_2_-ratio are presented in Figure 3. Non-pulmonary SOFA scores did not differ between these groups of baseline PaO_2_/FiO_2_-ratio (*P *= 0.389). Patients with a PaO_2_/FiO_2_-ratio lower than 300 mmHg (N = 356) had significantly higher plasma DNA concentrations compared to patients with a PaO_2_/FiO_2_-ratio higher than 300 mmHg (N = 199), median (IQR) 13,381 GE/mL (6,628 to 27,577 GE/mL) vs. 8,314 GE/mL (3,946 to 19,018 GE/mL) (*P *< 0.001), respectively. Concentrations did not differ between patients with ALI/ARDS (N = 47) and those without (N = 533): median (IQR) 13,707 GE/mL (7,700 to 35,596 GE/mL) vs. 11,562 GE/mL (5,171 to 24,130 GE/mL) (*P *= 0.06), respectively. Plasma DNA at admission correlated with a PaO_2_/FiO_2_-ratio at baseline (*P *< 0.001), baseline pH (*P *< 0.001), renal SOFA score at Day 1 (*P *< 0.001), and SAPS II score (*P *< 0.05), but not with tidal volume per predicted body weight (*P *= 0.47)

### Predictive value of plasma cell-free DNA

The area under the ROC curve (AUC) for plasma cell-free DNA concentration at admission as a predictor of 90-day mortality was 0.624 (95% CI 0.572 to 0.676); for the highest plasma cell-free DNA concentration the AUC was 0.605 (95% CI 0.552 to 0.657). The AUC was 0.643 (95% CI 0.581 to 0.704) in non-operative patients, 0.571 (95% CI 0.469 to 0.673) in post-operative patients, and 0.609 (95% CI 0.544 to 0.674) in patients with a baseline PaO_2_/FiO_2_-ratio lower than 300 mmHg. The best cut-off value for prediction of 90-day mortality was 16,000 GE/ml. Sensitivity, specificity and positive likelihood ratios of the best cut-off value in plasma cell-free DNA concentration at admission are presented in Table 3 with a comparison to the cut-off value from an earlier study [6].

**Table 3:** Comparison of two different cut-off points of plasma cell-free DNA for prediction of 90-day mortality

Cut-off point, GE/ml	**12,000 (Saukkonen 2008 **[6])	16,000 (present study)
Sensitivity	0.60 (0.53 to 0.68)	0.53 (0.45 to 0.60)
Specificity	0.55 (0.50 to 0.60)	0.69 (0.65 to 0.74)
Positive likelihood ratio	1.33 (1.13 to 1.57)	1.72 (1.40 to 2.11)
Odds ratio unadjusted	1.84 (1.28 to 2.65)	2.52 (1.31 to 4.83)
Odds ratio adjusted	1.39 (0.89 to 2.16)	2.22 (1.41 to 3.48)

Data (at admission) presented with 95% confidence intervals

GE, genome equivalents

In the first multivariate analysis, the following factors were independent predictors of 90-day mortality: plasma cell-free DNA concentration over 16,000 GE/ml at admission (*P *= 0.001), baseline PaO_2_/FiO_2_-ratio (*P *= 0.004), chronic heart disease (*P *< 0.001), and SAPS II score minus oxygenation (*P *< 0.001). The adjusted odds ratio (95% CI) for plasma cell-free DNA exceeding 16,000 GE/ml at admission was 2.22 (1.41 to 3.48) (Table 3). In the second multivariate analysis, plasma cell-free DNA divided into intervals of 5,000 GE/ml was a significant predictor of 90-day mortality (*P *= 0.001). Other variables found to be significant in this second multivariate analysis were the same as in the first analysis.

**Figure 4:**
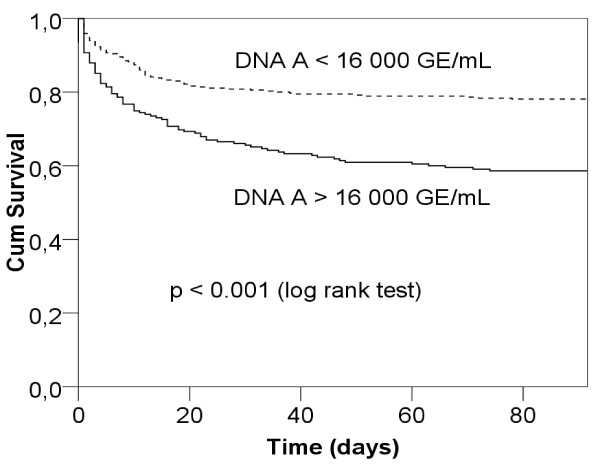
**Kaplan-Meier survival curves for 90-day mortality according to plasma cell-free DNA cut-off point at admission**. Sample A obtained at admission. GE, genome equivalents.

Kaplan-Meier survival curves for 90-day mortality according to the best cut-off value (16,000 GE/mL) are presented in Figure 4.

In the subgroup analysis of emergency patients with invasive ventilation exceeding 24 hours (N = 301), the median plasma cell-free DNA (IQR) at admission was 12,034 GE/ml (4,924 to 27,091 GE/ml) vs. 11,642 GE/ml (5,636 to 22,374 GE/ml) in other patients (N = 279) (*P *= 0.57). The AUC for the baseline plasma DNA as a predictor of 90-day mortality in this subgroup was 0.641 (95% CI 0.573 to 0.708).

## Discussion

To our knowledge, this is the largest multi-centre study evaluating plasma cell-free DNA concentrations in critically ill patients. We found that plasma cell-free DNA concentration at admission in mechanically ventilated patients was significantly higher in 90-day non-survivors than survivors. In addition, plasma cell-free DNA at admission over 16,000 GE/ml (and also divided in the increments of 5,000 GE/ml) was an independent predictor of 90-day mortality.

In previous studies, concentrations of plasma cell-free DNA have shown wide distribution. This variation may partly be explained by the use of multiple methods in DNA measurements. The problem is evident when exploring values reported in the control groups of healthy individuals [3]. The lack of a standardised method is also a reason why limits of normal values do not exist. Also, overlapping in cell-free DNA concentrations between healthy individuals and patients exists. For example, in the study of Rainer and colleagues, median plasma cell-free DNA concentration has been up to 1,170 GE/mL in patients with acute coronary syndrome [13], values being at the same level than measured in healthy individuals. In critically ill patients with severe sepsis or septic shock, a median plasma cell-free DNA concentration of 8,070 GE/mL was measured at admission [6], the analyses done in the same laboratory with the same method than in the present study. Patients with major trauma have had median plasma cell-free DNA concentrations over 181,000 GE/mL [9]. Kocsis and colleagues have found that levels of plasma cell-free DNA were significantly higher in severe acute pancreatitis than in the mild form of the same disease [8]. In their study, concentrations in the severe form of acute pancreatitis were high (median 989 ng/ml (149,848 GE/mL)) as were the concentrations in septic shock (median 3,303 ng/ml (500,454 GE/mL)). In the present study, the median concentration of plasma cell-free DNA at admission was 11,853 GE/mL, which was markedly lower than the levels in the Kocsis study [8].

In animal studies, isoprenaline has been shown to induce cell death and apoptosis in myocardial and skeletal myocytes *in vivo *in rats [25]. In clinical situations, adrenergic stress associated with critical illness itself and also catecholamines used may contribute to the release of DNA to circulation. Increased level of plasma DNA has been measured in healthy volunteers after excessive training, possibly reflecting an inflammatory reaction due to anaerobic metabolism [26]. We found a correlation between pH and plasma DNA at admission, a finding possibly reflecting the severity of illness and/or anaerobic tissue metabolism. A previous study has reported correlation between plasma cell-free DNA and SOFA score as a marker of multiple organ dysfunction and severity of illness [5]. Our results showed positive correlation between plasma DNA and SAPS II score and were consistent with that study. Thus, high levels of plasma cell-free DNA can simply be one expression of critical illness regardless of the specific diagnosis.

However, it is also possible that the observed high concentrations of plasma cell-free DNA in our study were caused by impaired oxygenation in particular. We found a correlation between the plasma cell-free DNA concentration and the severity of oxygenation problem (as reflected by the PaO_2_/FiO_2_-ratio) at study inclusion. In general, the lower was the PaO_2_/FiO_2_-ratio, the higher was the plasma cell-free DNA concentration. Moreover, non-pulmonary SOFA scores were comparable in different groups of PaO_2_/FiO_2_-ratio, suggesting the independent effect of impaired oxygenation on plasma cell-free DNA. This association has a physiological rationale as hypoxemia may cause tissue hypoxia and cell death. Unfortunately, we did not measure any other indicators of tissue hypoxia, for example, lactate concentration [27]. A previous study has shown a correlation between plasma cell-free DNA and lactate levels in patients with acute mesenteric ischemia, supporting the connection with tissue hypoxia and plasma DNA concentration [28].

Large tidal volumes are known to associate with the development of lung injury [15]. During the last decade, a lung protective ventilatory strategy with low tidal volumes (< 6 ml/kg) and plateau pressures (≤30 cmH_2_O) has been recommended in ALI/ARDS and sepsis [29]. In Finland, these recommendations have been adopted in practise only partly, since tidal volumes have been higher than recommended especially when calculated per predicted body weight (PBW) [17]. In the present study, we did not find a correlation between the plasma cell-free DNA concentration at admission and baseline tidal volume per predicted body weight. Thus, we could not confirm the association with large tidal volume and lung injury using plasma cell-free DNA as a surrogate of lung injury. However, the nature of the present study was observational and not designed to study this association. Neither did we find differences in plasma cell-free DNA concentrations between patients with or without ALI/ARDS. In theory, injured vascular endothelium and alveolar epithelium in ALI and ARDS could raise plasma cell-free DNA. The blood sampling (at admission and on Day 2) may have been too early to find the possible connection with ALI/ARDS, which often develops later. Instead, the concentration of plasma cell-free DNA at admission was significantly higher in patients with infection than without which is in accordance with earlier studies [5,6].

An earlier study found that cell-free DNA is rapidly eliminated from the blood [30]. On the other hand, plasma DNA concentrations have been found to be increased at 96 hours after the end of excessive training in healthy volun-teers, possibly associated with an ongoing inflammatory state [26]. In the present study, plasma samples were taken at two time points, at study inclusion and 36 hours later, and concentrations were at the same level in both samples. This observation may be explained by continuous release of DNA to circulation. The significance of renal function is not fully understood, but in the light of an earlier study, renal function should not affect concentrations [31]. In the present study, we found a correlation between renal SOFA score at Day 1 and plasma DNA at admission. However, in this study we cannot confirm or refute any causality between these factors.

In non-operative patients, concentrations of plasma cell-free DNA were higher in non-survivors than in survivors. In post-operative patients, there was no difference between non-survivors and survivors. One plausible explana-tion is that surgery *per se *may cause tissue injury that raises plasma cell-free DNA concentrations even when the disease process is not potentially life-threatening. In this study plasma cell-free DNA concentrations of post-operative patients were comparable to those of non-operative patients, despite a remarkably lower severity of illness (SAPS II score) of the post-operative patients. In addition, non-operative patients may have suffered their insult for a longer period of time ago than post-operative patients and, thus, plasma cell-free DNA may be more indicative of cell damage. Rising concentrations of plasma DNA in sample B in post-operative, but not in non-operative patients, suggests this possibility.

We found significantly higher plasma cell-free DNA concentrations at admission in 90-day non-survivors than survivors. A concentration over 16,000 GE/ml, and separately increments of 5,000 GE/ml, were independent predictors of 90-day mortality. However, the AUC for the prediction of 90-day mortality was poor. Furthermore, we found that sensitivity, specificity, and positive likelihood ratio were all lower when used in the group of mechanically ventilated patients than in sepsis patients [6]. In some earlier studies, the predictive value of plasma cell-free DNA has been better [5,9,10,13]. However, in these studies follow-up times have varied and the patient numbers may have been inadequate for the prediction of mortality.

### Limitations

Our study has some limitations. First, our cohort of patients was inevitably heterogeneous. As a result, it is possible that some underlying conditions, (for example, occult neoplasia, pregnancy, autoimmune disorders, trauma), may have had an unexpected impact on plasma cell-free DNA values. This heterogeneity is, however, the situation in real life. In addition, the results from the analysis of the subgroup with more severely impaired oxygenation showed that more strict criteria did not change the results confirming the robustness of our findings. Second, we measured plasma DNA only at two points, both presumably near the onset of disease. More remote sampling would possibly have provided information about the association to development of ARDS and the healing process. However, our aim was to specifically concentrate on prog-nostication, and, thus, early sampling independent of clinical treatment choices is essential. Finally, the present study is ob-servational by its nature and, thus, could not confirm or refute any causalities.

## Conclusions

In conclusion, we found that plasma cell-free DNA values are commonly high in mechanically ventilated patients. Concentrations measured at admission were higher in 90-day non-survivors than survivors, and predicted indepen-dently 90-day mortality. However, an increased plasma cell-free DNA concentration is neither a sensitive nor a specific predictor of death. Thus, the clinical usefulness of this test seems to be limited.

## Key messages

• Plasma cell-free DNA is commonly increased in patients needing mechanical ventilation.

• Plasma cell-free DNA levels are higher in 90-day non-survivors than survivors.

• However, the predictive value of 90-day mortality in mechanically ventilated patients seems to be poor.

## Abbreviations

ACS: acute coronary syndrome; AECC: American-European Consensus Conference; ALI: acute lung injury; APACHE: Acute Physiology and Chronic Health evaluation; ARDS: acute respiratory distress syndrome; GE: genome equivalent; ICD10: International Statistical Classification of Diseases and Related Health Problems; MV: mechanical ventilation; SAPS: Simplified Acute Physiology Score; SOFA: Sequential Organ Failure Assessment

## Competing interests

The authors declare that they have no competing interests.

## Authors' contributions

MO participated in the study design and data collection, analysed data, and drafted and revised the manuscript. PL carried the responsibility for the laboratory method and analysis, and participated in manuscript writing. AMK, IP and MR were involved in data collection and helped in the manuscript writing. TV conceived the study design and helped in the manuscript writing. VP conceived the study design, advised in the statistical analyses and in the manuscript writing. All authors have contributed intellectually to the content of the manuscript and read and approved the final version.
